# Cow's milk protein allergy in children: a practical guide

**DOI:** 10.1186/1824-7288-36-5

**Published:** 2010-01-15

**Authors:** Carlo Caffarelli, Francesco Baldi, Barbara Bendandi, Luigi Calzone, Miris Marani, Pamela Pasquinelli, on behalf of EWGPAG

**Affiliations:** 1 Dipartimento dell'Età Evolutiva, Clinica Pediatrica Università di Parma, Parma, Italy; 2 UO di Pediatria, AUSL Imola, Imola, Italy; 3 Dipartimento "Salute della donna, del bambino e dell'adolescente" Policlinico S Orsola-Malpighi, Clinica Pediatrica, Bologna, Italy; 4 Dipartimento Emergenza ed Accettazione diagnostica, UO di Pediatria, Fidenza, Italy; 5 Pediatria, AUSL di Ravenna, Italy; 6 UO Pediatria, AUSL di Cesena, Italy

## Abstract

A joint study group on cow's milk allergy was convened by the Emilia-Romagna Working Group for Paediatric Allergy and by the Emilia-Romagna Working Group for Paediatric Gastroenterology to focus best practice for diagnosis, management and follow-up of cow's milk allergy in children and to offer a common approach for allergologists, gastroenterologists, general paediatricians and primary care physicians.

The report prepared by the study group was discussed by members of Working Groups who met three times in Italy. This guide is the result of a consensus reached in the following areas. Cow's milk allergy should be suspected in children who have immediate symptoms such as acute urticaria/angioedema, wheezing, rhinitis, dry cough, vomiting, laryngeal edema, acute asthma with severe respiratory distress, anaphylaxis. Late reactions due to cow's milk allergy are atopic dermatitis, chronic diarrhoea, blood in the stools, iron deficiency anaemia, gastroesophageal reflux disease, constipation, chronic vomiting, colic, poor growth (food refusal), enterocolitis syndrome, protein-losing enteropathy with hypoalbuminemia, eosinophilic oesophagogastroenteropathy. An overview of acceptable means for diagnosis is included. According to symptoms and infant diet, three different algorithms for diagnosis and follow-up have been suggested.

## Introduction

Cow's milk protein allergy (CMPA) affects from 2 to 6% of children, with the highest prevalence during the first year of age [1]. About 50% of children have been shown to resolve CMPA within the first year of age, 80-90% within their fifth year [2,3]. The rate of parent-reported CMPA is about 4 times higher than the real one in children [4]. So, many children are referred for suspected CMPA based on parent perception, symptoms such as cutaneous eruption, insomnia, persistent nasal obstruction, sebhorreic dermatitis or positive results to unorthodox investigations. Moreover, parents often put their children on unnecessary diet without an adequate medical and dietary supervision. These inappropriate dietary restrictions may provoke nutritional unbalances, especially in the first year of age. Therefore, an accurate diagnosis of CMPA is important in order to avoid not only the risk of rickets, decreased bone mineralization [5], anaemia, poor growth and hypoalbuminemia, but also that of immediate clinical reactions or severe chronic gastroenteropathy leading to malabsorption.

Recently, three guidelines [6-8] reporting different approaches to the infant with CMPA have been published.

In view of these considerations, a study group with expert representatives of Emilia-Romagna Working Group for Paediatric Allergy and of that for Paediatric Gastroenterology (EWGPGA), was constituted. As mmembers of the expert panel, the authors were assigned to review practice with regard to diagnosis, management and follow-up of CMPA for both community and hospital paediatrician in order to share the same approach towards the child. The document prepared by the study group was based on existing recommendations, clinical experience and evidence from the literature. The report was discussed and received input by the members (see participant list in acknowledgments) of EWGPGA which included clinicians experienced in paediatric allergy, paediatric gastroenterology and general paediatricians, in three meetings held in November 2008, February 2009 and March 2009 and a consensus was reached. According to the symptoms and the type of infant diet, three different algorithms for diagnosis and follow-up have been suggested. These approaches refer to the child in the first year of age. Recommendations for older children have been briefly reported.

## Cow's milk protein allergy: when should we doubt?

**Figure 1:**
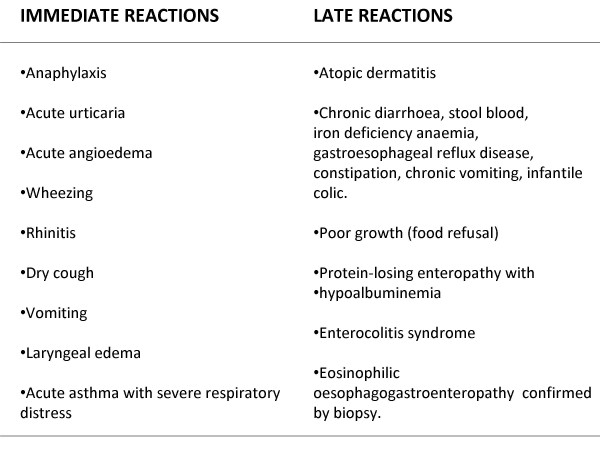
**Immediate and late onset reactions in children with cow's milk protein allergy**.

A positive atopic familiar history is common in children with suspected CMPA [9]. The diagnosis of CMPA is based on a detailed history of symptoms (Fig. 1), skin prick test and serum specific IgE to cow's milk protein, elimination diet and oral food challenge. Clinical manifestations due to CMPA [6-14] can be divided into IgE-mediated immediate clinical reactions (onset of the symptoms within the 30 minutes after the ingestion of cow's milk) and non IgE-mediated delayed reactions (hours-days after food ingestion), most affecting the skin and the gastrointestinal system. However, immediate and delayed reactions can be associated in atopic eczema and in eosinophilic oesophageal gastroenteritis (Fig. 1).

The negative predictive value of skin prick test/specific IgE for immediate reaction is excellent (>95%) [15], however a small number of these patients can have clinical reaction. Therefore, despite negative IgE tests if there is a strong suspicion of CMPA, an oral food challenge is necessary to confirm the absence of clinical allergy. On the other hand, about 60% of children with positive IgE tests have CMPA [15,16]. Prick by prick test with cow's milk substitutes may be considered.

Oral food challenge, open or blind, remains the 'gold standard" to definitely ascertain children with food allergy when the diagnosis is unclear [17]. OFC should be performed under medical supervision in a setting with emergency facilities, especially in case of positive skin prick test or serum specific IgE to cow's milk and in infants at risk of an immediate reaction.

## Cow's milk substitutes

About 10% of children with CMPA react to extensively hydrolyzed formula (eHF) [7]. In comparison with eHF, soy formula (SF) provokes more frequently reactions in children with CMPA aged less than 6 months [18] but not in older children. SF mainly induces gastrointestinal symptoms.

Amino acid formula (AAF) is non allergenic [19]. Its use is limited by the high cost and bad taste.

Rice is allergenic and is often involved in the onset of enterocolitis syndrome in Australian infants [20]. Contrasting data have been reported on the effect of protein content on growth [21]. In Italian children, rice formula has been shown to be tolerated by children with CMPA [22]. Larger long-term studies are warranted to clarify the use of rice formula in infants with CMPA. Rice formula may be a choice in selected cases taking into consideration the taste and the cost.

Home-made meals may be a dietary option after 4 months of age.

Mammalian milks are not nutritionally adequate. Goat's milk commonly provokes clinical reactions in more than 90% of children with CMPA [23], donkey's milk in about 15% [24,25] and has a high cost.

## A child fed with cow's milk formula with mild- moderate symptoms (Fig. 2)

**Figure 2:**
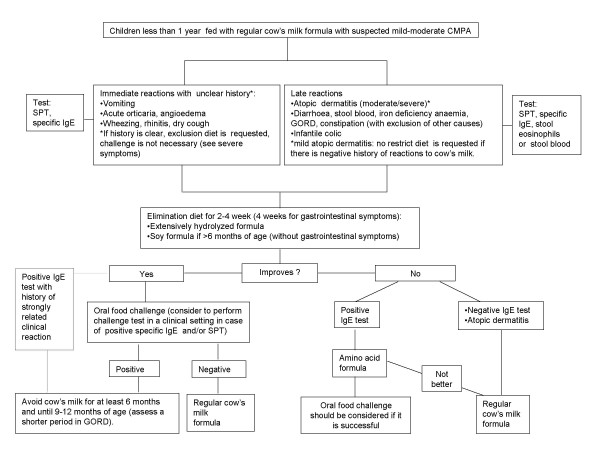
**Algorithm for children < 1 year fed with cow's milk formula and mild-moderate symptoms**.

In infants with immediate symptoms (vomiting, acute hives, angioedema, wheezing, rhinitis, dry cough) or late symptoms (moderate/severe atopic dermatitis, diarrhoea, blood in the stools, iron deficiency anaemia, gastroesophageal reflux disease (GORD), constipation) a CMPA can be suspected [6-8,10-14]. Other causes are to be considered for patients unresponsive to treatment. Infant colic (more than 3 hours of crying a day, 3 days for more than 3 weeks) is not unanimously considered as a consequence of CMPA. The paediatrician has to consider the opportunity of a cow's milk free diet in the most troublesome cases [26,27]. Mild immediate reactions may be of difficult interpretation because they can be the result of causes different from CMPA. However, if these symptoms are strongly related to cow's milk ingestion, we recommend to eliminate cow's milk and follow the algorithm for severe reactions (Fig. 2).

Regarding delayed onset gastrointestinal symptoms, other pathologies (i.e. infections) should be excluded before investigating allergic sensitization.

In mild atopic dermatitis, investigations for CMPA are not necessary in the absence of a clear relation between cow's milk intake and onset of symptoms.

When a CMPA is suspected, infants should go on a 2-4 week diet without cow's milk protein. Four weeks should be considered for chronic gastrointestinal symptoms. Infants should be fed with eHF or SF in children aged more than 6 months and without gastrointestinal symptoms.

If the symptoms improve on a restrict diet, an OFC to cow's milk is necessary to definitely ascertain the diagnosis. If the oral food challenge is positive, the child must follow the elimination diet and can be re-challenged after 6 months (a shorter period for GORD) and in any case, after 9-12 months of age. If the oral food challenge is negative, a free-diet can be followed.

When there is strong suspicion of IgE-mediated reactions, in infants who do not respond to a diet with eHF or SF an attempt may be made with a 14-days diet with AAF.

Cow's milk substitutes are used in children aged less than 12 months. In older children with CMPA, eHF or AAF are not usually necessary because an adequate diet is easily accessible.

## A child fed with cow's milk formula with severe symptoms (Fig. 3)

**Figure 3:**
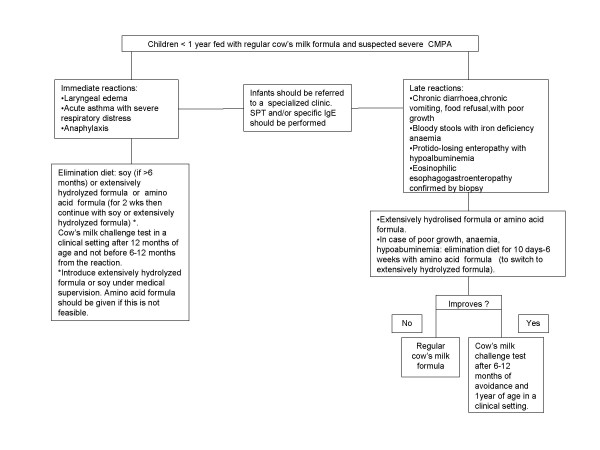
**Algorithm for children <1 year fed with cow's milk formula and severe symptoms**. In child less than1 year of age, infant formula is not compulsory.

Immediate severe symptoms are considered laryngeal edema, acute asthma with severe respiratory difficulty, anaphylaxis. The following are delayed onset severe symptoms: chronic diarrhoea or chronic vomiting with poor growth, intestinal bleeding with iron deficiency anaemia, protein losing enteropathy with hypoalbuminemia, eosinophilic gastroenteropathy confirmed by biopsy [7,8,10-14].

If any of these immediate symptoms are observed as a consequence of suspected CMPA, infants should follow a cow's milk free diet. As substitutes, SF (if older than 6 months of age) or eHF or AAF can be used. eHF and SF should be started under medical supervision because of possible clinical reactions. If an AAF is adopted, it may be administered for 2 weeks and then the infant may be switched to SF or eHF.

In children with late severe gastrointestinal symptoms with poor growth, anaemia or hypoalbuminemia or eosinophilic oesophagogastroenteropathy, it is recommended to start an elimination diet with AAF and then switched with eHF. The effect of the diet should check out within 10 days for enterocolitis syndrome, 1-3 weeks for enteropathy and 6 weeks for eosinophilic oesophagogastroenteropathy.

In children with anaphylaxis and concordant positive IgE tests or severe gastrointestinal reactions, oral food challenge is not necessary for diagnosis. The oral food challenge for tolerance acquisition should be performed not before 6-12 months after the last reaction. Children have to eliminate cow's milk until 12 months of age, but in those with enterocolitis syndrome until 2-3 years of age [28].

Children with any severe symptoms should be referred to a specialized centre.

eHF or AAF is used in children aged less than 12 months and in older children with severe gastrointestinal symptoms. In children > 12 months with anaphylaxis, cow's milk substitutes are not always nutritionally required.

## A breast-fed infant with a suspected CMPA (Fig. 4)

**Figure 4:**
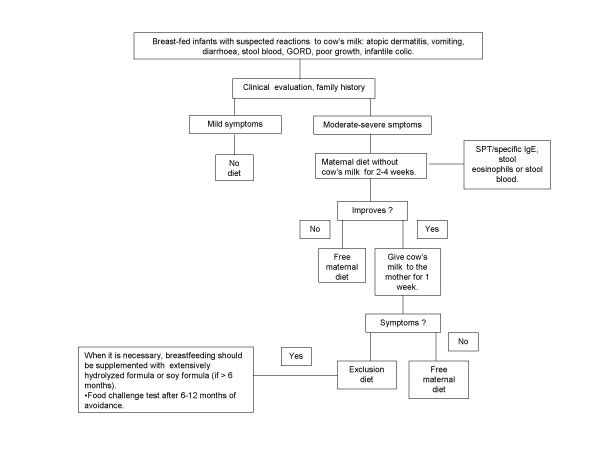
**Algorithm for breast-fed infants with suspected non-IgE mediated reactions to cow's milk protein**.

In exclusively breast-fed infants, suspected symptoms to the cow's milk proteins are almost always non IgE-mediated as atopic dermatitis, vomiting, diarrhoea, blood in the stools, GORD, colic [29].

A maternal diet without cow's milk is not recommended for mild symptoms.

There is no evidence that a maternal diet without egg and cow's milk in infants with bloody stools (proctocolitis) is of value [30,31].

In infants with moderate-severe symptoms, cow's milk protein, eggs and other foods should be eliminated by the mother's diet only if history suggests an unequivocal reaction. Moreover, the infant should be referred to a specialized centre. The maternal elimination diet has to be followed for 4 weeks. If there is no improvement the diet should be stopped. If symptoms improved, it's recommended that the mother ingested large amounts of cow's milk for one week. If symptoms occurred, the mother will continue the diet with supplemental intake of calcium. The infant can be weaned as recommended for healthy children, but cow's milk should be avoided until 9-12 months of age and for at least 6 months from the beginning of the diet. If the volume of breast milk is insufficient, eHF or SF formula (if > 6 months) should be administered.

If after the reintroduction of cow's milk in mother's diet symptoms do not occur, the excluded foods can be introduced one by one in the diet.

## Conclusions

The diagnosis of CMPA is based on oral food challenge that follows a 2-4 week elimination diet.

A diagnostic oral food challenge is unnecessary in immediate reactions or late gastrointestinal reactions with anaemia, poor growth or hypoalbuminemia if the causative role of cow's milk is clear. Children can be challenged after 6-12 months from the reaction and not before 12-24 months of age according to the symptoms.

Diets must be nutritionally balanced. In children with CMPA, a supplementation with calcium must be evaluated.

Diet is not requested in children with mild atopic dermatitis and negative history for cow's milk reactions.

SF should not be used in infants < 6 months of age with allergic symptoms and in those with late gastrointestinal symptoms.

Children with gastrointestinal reactions and anaemia, poor growth or hypoalbuminemia should be given AAF and then switched to eHF.

eHF or AAF is used in children aged less than 12 months and in older children with severe gastrointestinal symptoms. In children > 12 months with anaphylaxis, cow's milk substitutes are not always nutritionally required.

## List of abbreviations

CMPA: cow's milk protein allergy; EWGPAG: Emilia-Romagna Working Group for Paediatric Allergy and of that for Paediatric Gastroenterology; eHF: extensively hydrolyzed formula; SF: soy; AAF: amino acid formula; GORD: gastroesophageal reflux disease.

## Competing interests

The authors declare that they have no competing interests.

## Authors' contributions

CC, FB, BB, LC, MM, PP conceived the design of the study and participated in its coordination. They prepared the draft of the manuscript and revised it. All authors read and approved the final manuscript.

## References

[B1] Host A Frequency of cow's milk allergy in childhood Ann Allergy Asthma Immunol 2002 89 6 Suppl 1 33 7 12487202 10.1016/s1081-1206(10)62120-5

[B2] Wood RA The natural history of food allergy Pediatrics 2003 111 1631 1637 12777603

[B3] Host A Halken S Jacobsen HP Christensen AE Herskind AM Plesner K Clinical course of cow's milk protein allergy/intolerance and atopic diseases in childhood Pediatr Allergy Immunol 2002 13 Suppl 15 23 28 10.1034/j.1399-3038.13.s.15.7.x 12688620

[B4] Rona RJ Keil T Summers C Gislason D Zuidmeer L Sodergren S Sigurdardottir T Lindner T Goldhahn K Dahlstrom J The prevalence of food allergy A meta-analysis J Allergy Clin Immunol 2007 120 638 46 10.1016/j.jaci.2007.05.026 17628647

[B5] Black RE Williams SM Jones IE Goulding A Children who avoid drinking cow milk have low dietary calcium intakes and poor bone health Am J Clin Nutr 2002 76 675 680 12198017 10.1093/ajcn/76.3.675

[B6] Bhatia J Greer F the Committee on Nutrition Use of Soy Protein-Based Formulas in Infant Feeding Pediatrics 2008 121 1062 1068 10.1542/peds.2008-0564 18450914

[B7] Vandenplas Y Brueton M Dupont C Hill D Isolauri E Koletzko S Oranje AP Staiano A Guidelines for the diagnosis and management of cow's milk protein allergy in infants Arch Dis Child 2007 92 902 908 10.1136/adc.2006.110999 17895338 PMC2083222

[B8] Kemp AS Hill DJ Allen KJ Anderson K Davidson GP Day AS Heine RG Peake JE Prescott SL Shugg AW Sinn J Guidelines for the use of infant formulas to treat cows milk protein allergy: an Australian consensus panel opinion MJA 2008 188 109 112 18205586 10.5694/j.1326-5377.2008.tb01534.x

[B9] Greer FR Sicherer SH Wesley Burks A the Committee on Nutrition and Section on Allergy and Immunology Effects of early nutritional interventions on the development of atopic disease in infants and children: the role of maternal dietary restriction, breastfeeding, timing of introduction of complementary foods and hydrolyzed formulas Pediatrics 2008 121 183 191 10.1542/peds.2007-3022 18166574

[B10] Host A Cow's milk protein allergy and intolerance in infancy. Some clinical, epidemiological and immunological aspects Pediatr Allergy Immunol 1994 5 5 Suppl 1 36 7704117

[B11] Heine RG Elsayed S Hosking CS Hill DJ Cow's milk allergy in infancy Curr Opin Allergy Clin Immunol 2002 2 217 25 10.1097/00130832-200206000-00011 12045418

[B12] Salvatore S Vandenplas Y Gastroesophageal reflux and cow milk allergy: is there a link? Pediatrics 2002 110 972 84 10.1542/peds.110.5.972 12415039

[B13] Iacono G Cavataio F Montalto G Intolerance of cow's milk and chronic constipation in children N Engl J Med 1998 399 1100 1104 10.1056/NEJM199810153391602 9770556

[B14] Simeone D Miele E Boccia G Marino A Troncone R Staiano A Prevalence of atopy in children with chronic constipation Arch Did Child 2008 93 1044 1047 10.1136/adc.2007.133512 18562455

[B15] Celik-Bilgili S Mehl A Verstege A The predictive value of specific immunoglobulin E levels in serum for the outcome of oral food challenges Clin Exp Allergy 2005 35 268 73 10.1111/j.1365-2222.2005.02150.x 15784102

[B16] Verstege A Mehl A Rolinck-Werninghaus C Staden U Noconw M Beyer K Niggemann B The predictive value of the skin prick test weal size for the outcome of oral food challenges Clin Exp Allergy 2005 35 1220 1226 10.1111/j.1365-2222.2005.2324.x 16164451

[B17] Bock SA Sampson HA Atkins FM Zeiger RS Lehrer S Sachs M Bush RK Metcalfe DD Double-blind placebo-controlled food challenge (DBPCFC) as an office procedure: a manual J Allergy Clin Immunol 1988 82 986 97 10.1016/0091-6749(88)91021-4 3060514

[B18] Klemola T Vanto T Juntunen-Backman K Kalimo K Korpela R Varjonen E Allergy to soy formula and to extensively hydrolyzed whey formula in infants with cow's milk allergy: A prospective, randomized study with a follow-up to the age of 2 years J Ped 2002 140 219 24 10.1067/mpd.2002.121935 11865274

[B19] Hill DJ Murch SH Rafferty K Wallis P Green JC The efficacy of amino acid-based formulas in relieving the symptoms of cow's milk allergy: a systematic review Cl Exp Allergy 2007 37 808 822 10.1111/j.1365-2222.2007.02724.x 17517094

[B20] Mehr SS Kakakios AM Kemp AS Rice: a common and severe cause of food protein-induced enterocolitis syndrome Arch Dis Child 2009 94 220 223 10.1136/adc.2008.145144 18957470

[B21] Lasekan JB Koo WKW Walters J Neylan M Luebbers S Growth, tolerance and biochemical measures in healthy infants fed a partially hydrolyzed rice protein-based formula: a randomized, blinded, prospective trial Journ Am Coll Nutr 2006 25 12 19 10.1080/07315724.2006.10719509 16522927

[B22] Fiocchi A Restani P Bernardini R Lucarelli S Lombardi G Magazzu G Marseglia GL Pittschieler K Tripodi S Troncone R Ranzini C Hydrolysed rice-based formula is tolerated by children with cow's milkallergy: a multi-centre study Cl Exp All 2006 36 311 316 10.1111/j.1365-2222.2006.02428.x 16499641

[B23] Bellioni-Businco B Paganelli R Lucenti P Giampietro PG Perborn H Businco L Allergenicity of goat's milk in children with cow's milk allergy J Allergy Clin Immunol 1999 103 1191 1194 10.1016/S0091-6749(99)70198-3 10359905

[B24] Monti G Bertino E Muratore MC Coscia A Cresi F Silvestro L Fabris C Fortunato D Giuffrida MG Conti A Efficacy of donkey's milk in treating highly problematic cow's milk allergic children: an in vivo and in vitro study Pediatr Allergy Immunol 2007 18 258 264 10.1111/j.1399-3038.2007.00521.x 17433003

[B25] Tesse R Paglialunga C Braccio S Armenio L Adequacy and tolerance to ass's milk in an Italian cohort of children with cow's milk allergy Ital J Pediatr 2009 35 19 10.1186/1824-7288-35-19 19589131 PMC2717565

[B26] Heine RG Allergic gastrointestinal motility disorders in infancy and early childhood Pediatr Allergy Immunol 2008 19 383 391 10.1111/j.1399-3038.2008.00785.x 18713339

[B27] Savino F Focus on infantile colic Acta Paediatr 2007 96 1259 1264 10.1111/j.1651-2227.2007.00428.x 17718777

[B28] Nowak-Wegrzyn A Sampson HA Wood RA Sicherer SH Food protein-induced enterocolitis syndrome caused by solid food proteins Pediatrics 2003 111 829 35 10.1542/peds.111.4.829 12671120

[B29] Hill DJ Roy N Heine RG Hosking CS Francis DE Brown J Speirs B Sadowsky J Carlin JB Effect of a low-allergen maternal diet on colic among breastfed infants: a randomized, controlled trial Pediatrics 2005 116 e709 e715 10.1542/peds.2005-0147 16263986

[B30] Xanthakos SA Schwimmwe JB Melin-Aldana H Rothemberg ME Witte DP Cohen MB Prevalence and outcome of allergic colitis in healthy infants with rectal bleeding: A prospective cohort study J Pediatr Gastroenterol Nutr 2005 41 16 22 10.1097/01.MPG.0000161039.96200.F1 15990624

[B31] Arvola T Ruuska T Keranen J Hyoty H Salminen S Isolauri E Rectal bleeding in infancy: clinical, allergological and microbiological examination Pediatrics 2006 117 e760 e768 10.1542/peds.2005-1069 16585287

